# Role of home-based pulmonary rehabilitation programs for disease progression and quality of life in patients with stable bronchiectasis: a single-center RCT

**DOI:** 10.3389/fmed.2025.1571837

**Published:** 2025-07-16

**Authors:** Siwen Min, Hongting Liu, Yajuan Zhang, Jingyi Wang, Li Wang, Yifan Jiang, Suxia Shi

**Affiliations:** ^1^Department of Nursing, Shanghai Pulmonary Hospital Affiliated to Tongji University, Shanghai, China; ^2^Department of Nursing, Shanghai Sixth People's Hospital Affiliated to Shanghai Jiao Tong University, Shanghai, China; ^3^School of Medicine, Tongji University School of Medicine, Shanghai, China; ^4^Department of Nursing, Shanghai Tenth People’s Hospital Affiliated to Tongji University, Shanghai, China; ^5^Department of Nursing, Shanghai General Hospital Affiliated to Shanghai Jiao Tong University, Shanghai, China

**Keywords:** bronchiectasis, home-based pulmonary rehabilitation, quality of life, clinical outcomes, nursing

## Abstract

**Objective:**

This study aims to evaluate the effectiveness of a home-based pulmonary rehabilitation program for patients with stable bronchiectasis in improving their quality of life and reducing acute exacerbations.

**Methods:**

A randomized controlled trial design was employed, with patients randomly assigned to an intervention group (receiving the pre-designed home-based pulmonary rehabilitation program for bronchiectasis) or a control group (receiving standard respiratory care). The effects of the program on quality of life, lung function indicators, and the frequency of acute exacerbations were assessed.

**Results:**

Preliminary findings indicate that the home-based pulmonary rehabilitation program significantly improved quality of life scores, enhanced lung function, and reduced the frequency of acute exacerbations (*p* < 0.05).

**Conclusion:**

The home-based pulmonary rehabilitation program is an effective intervention for patients with stable bronchiectasis, contributing to improved clinical outcomes and quality of life.

## 1 Introduction

Bronchiectasis is a chronic respiratory disease caused by multiple etiologies, characterized by irreversible bronchial dilation accompanied by airway inflammation and recurrent infections. In recent years, the prevalence of this condition has shown a global upward trend. A large-scale cross-sectional survey conducted in urban areas of seven provinces and cities in China, including Beijing, Shanghai, and Guangdong, reported a physician-diagnosed prevalence of bronchiectasis of 1.2% among residents aged 40 years and older, with a significant increase observed with advancing age (1). Previous studies have indicated that the disease is more common in females and that its incidence rises markedly with age. Due to its chronic and relapsing nature, patients often require long-term home management even after symptom relief and hospital discharge. However, it has been reported that approximately 35.61 to 52.39% of patients fail to receive adequate long-term management and effective home monitoring, resulting in persistent airway inflammation, progressive structural damage, and a significant decline in quality of life (1). Poor disease control may further accelerate disease progression and increase the risk of developing chronic obstructive pulmonary disease (COPD) through ongoing airway inflammation, recurrent infection-induced tissue destruction, and airway remodeling (2).

Home-based Pulmonary Rehabilitation (HBPR), owing to its personalized design, ease of implementation, and low resource consumption, has gradually emerged as an attractive tele-rehabilitation model. HBPR typically includes individualized breathing training (such as abdominal and pursed-lip breathing), regular walking or light aerobic exercises, and airway clearance techniques (such as postural drainage and active cycle of breathing techniques) (3). Previous studies have confirmed that HBPR can significantly improve exercise tolerance, alleviate dyspnea, and enhance quality of life in patients with COPD (4). However, research on the application of HBPR in patients with bronchiectasis remains limited, and its potential in improving pulmonary function, exercise tolerance, and quality of life requires further verification.

This study aims to evaluate the therapeutic effect of HBPR in patients with bronchiectasis by assessing its impact on improving pulmonary function, enhancing exercise capacity, relieving symptoms, and improving quality of life (3, 4). By comparing its efficacy with conventional treatment, this study seeks to provide a novel intervention strategy for the long-term management of bronchiectasis, especially expanding daily management possibilities for patients in the stable phase and promoting the broader clinical adoption of HBPR in chronic respiratory diseases.

## 2 Subjects and methods

### 2.1 Research subjects

Patients with bronchiectasis in the stable phase who were discharged from the Department of Respiratory Medicine of Shanghai Pulmonary Hospital between October 2023 and December 2023 were included in this study and met the following criteria.

#### 2.1.1 Inclusion criteria

(1) Clearly diagnosed with bronchiectasis in the stable phase (5) (respiratory symptoms did not exceed the normal daily fluctuation range within the recent 4 weeks); (2) Clinical manifestations were in line with the characteristics of bronchiectasis; (3) Bronchiectasis was confirmed by high-resolution chest computed tomography (CT); (4) Able to complete pulmonary function tests; (5) Signed the informed consent form; and (6) Aged 18 years or older and able to use smartphones or WeChat.

#### 2.1.2 Exclusion criteria

(1) Complicated with severe organic diseases; (2) Having significant limb dysfunction; (3) Suffering from mental or intellectual disorders; (4) Participating in other pulmonary rehabilitation programs; and (5) Other situations where it is impossible to cooperate with rehabilitation training.

As this was a preliminary exploratory study, the sample size was determined based on feasibility, including expected patient volume and site capacity during the study period. This approach was consistent with similar pilot studies and aimed to generate initial evidence for future formal sample size calculations.

### 2.2 Research methods

#### 2.2.1 Research design

This study adopted a randomized controlled trial (RCT) design to evaluate the effectiveness of an evidence-based home-based pulmonary rehabilitation program for patients with stable bronchiectasis (6). Eligible hospitalized patients were randomly assigned to either the intervention group or the control group at a 1:1 ratio using a computer-generated randomization sequence.

To enhance the scientific rigor of the study and minimize bias, a single-blind design was employed. Random group allocation was performed by the study coordinator, and participants were blinded to their group assignments. The intervention was delivered by specially trained nursing staff, while data collection and outcome assessment were conducted by an independent team of blinded researchers to avoid interaction between implementers and evaluators.

In addition, to ensure the reliability of the data, standardized tools (such as pulmonary function assessment forms and quality-of-life questionnaires) and uniform record templates were used to measure and document all outcome indicators consistently during the follow-up period.

To minimize attrition bias, several retention strategies were implemented, including adherence screening before enrollment, weekly telephone or video follow-ups, real-time feedback collection through electronic logs, and technical support to ensure sustained participation. Notably, all enrolled participants completed the 12-month follow-up, with no dropouts, thus ensuring the completeness and integrity of outcome analysis.

#### 2.2.2 Intervention group

The intervention group implemented an evidence-based home-based pulmonary rehabilitation program, aiming to improve patients’ pulmonary function, enhance exercise tolerance, relieve symptoms and improve the quality of life through multi-dimensional intervention measures. The specific intervention contents include the following aspects.

(1) Exercise training: Patients in the intervention group carried out lower limb endurance training for 30–60 min every day. The main forms included walking, cycling or other light to moderate intensity aerobic exercises. The exercise intensity was individually adjusted according to the target heart rate range or the modified Borg dyspnea score (with a target score of 3–4 points). Based on the patients’ physical conditions, the training intensity and duration were gradually increased. Meanwhile, specialized exercise instructors provided remote or face-to-face guidance every week to ensure the safety and effectiveness of the patients’ training. (2) Respiratory training: Combine inspiratory muscle training with conventional pulmonary rehabilitation techniques to enhance the strength and endurance of the respiratory muscle groups. For inspiratory muscle training, a dedicated inspiratory muscle trainer [such as Threshold IMT (Inspiratory Muscle Training)] is used. The initial intensity is set at 30–50% of the maximum inspiratory pressure (PImax). Each training session lasts for 10–15 min and is conducted twice a day, with the intensity gradually increased. Conventional pulmonary rehabilitation includes abdominal breathing and pursed-lip breathing to improve the alveolar ventilation efficiency of patients and relieve dyspnea. (3) Airway clearance: Patients are taught individualized airway clearance techniques by specially trained respiratory therapists, and specific plans are adjusted according to the specific conditions and tolerance of the patients. Common methods include: Active Cycle of Breathing Techniques (ACBT): By combining controlled breathing, deep inhalation and forced exhalation to promote the discharge of sputum; Postural drainage: Select appropriate postures according to the distribution of patients’ sputum to assist with drainage, and combine gentle back patting to enhance the effect. (4) Health education: Weekly push content related to disease management and lifestyle adjustments for bronchiectasis through WeChat or other e-health platforms, including reasonable dietary suggestions, emotion management strategies, guidance on medication adherence and specific measures to cope with acute exacerbations. Patients can also consult specialist nurses through online Q&A platforms to obtain personalized health advice, so as to enhance the initiative and scientific nature of home management. (5) Nutritional management: It includes assessment, nutritional intake and supplementation, nutritional support and improvement of compliance. (6) Quality management: It is mainly completed under the supervision of nurses, including remote follow-up and training guidance, vital sign monitoring and oxygen therapy management, as well as improvement of training safety and compliance.

In addition, in order to ensure the intervention effect and patient compliance, a detailed follow-up plan was formulated. The nursing team would conduct telephone or video follow-ups every week to assess changes in patients’ symptoms, the effect of airway clearance and the completion of exercise training, and adjust the intervention plan when necessary. Patients were also required to record the completion of exercise training, airway clearance and health education on a daily basis and submit feedback regularly through electronic logs.

#### 2.2.3 Control group

The control group received routine respiratory care, aiming to ensure that patients received standardized disease:

1. Distribution of health education manuals: When patients were discharged from the hospital, nursing staff distributed health education manuals on bronchiectasis to them. The contents of the manuals included basic knowledge of the disease, coping strategies for common symptoms, methods of using medications, suggestions for lifestyle adjustments (such as diet and exercise), and measures to prevent acute exacerbations. Through a simple and easy-to-understand format, it helped patients and their families understand the importance of disease management and improve their health literacy. When discharged from the hospital, patients received the health education manuals compiled by the professional team of the Department of Respiratory Medicine. The contents of the manuals covered the basic knowledge of bronchiectasis, common symptoms and their management strategies, guidance on medication use, suggestions for lifestyle adjustments (such as diet and exercise), and methods to prevent acute exacerbations. Patients can obtain comprehensive disease management information through the manuals to improve their self-management ability and health literacy.
2. Push of rehabilitation education videos and official accounts: After being discharged from the hospital, patients could obtain the push of rehabilitation education videos and disease management content every week through the hospital’s WeChat official account or relevant online platforms. The videos were recorded by professional teams, and the contents covered the following aspects. ① Key points for the long-term management of bronchiectasis. ② Basic respiratory training (such as abdominal breathing and pursed-lip breathing). ③ Dietary guidance and daily care strategies. ④ Suggestions for the early identification and treatment of acute exacerbations. These pushes aimed to help patients master the key knowledge of disease management in a scientific and practical manner and provide continuous educational support.
3. Regular follow-up examinations: Patients in the control group underwent regular follow-up examinations as planned after being discharged from the hospital (such as at the 4th week and the 12th week). A comprehensive follow-up assessment was completed by the respiratory chronic disease nursing team. The contents of the follow-up examinations included: ① Recording changes in clinical symptoms (such as cough frequency, sputum volume and color changes); ② Measurement of pulmonary function indicators (such as FEV₁, FVC); ③ Evaluation of quality of life (such as through the Quality of Life in Bronchiectasis (QoL-B) questionnaire specific to bronchiectasis); ④ Feedback and guidance on patients’ medication adherence and the implementation of rehabilitation; ⑤ The results of the follow-up examinations would be used to optimize the subsequent management plans for patients, and it was recommended to adjust the treatment plans when necessary.
4. Routine health management support: Besides regular follow-up examinations, patients could obtain continuous support services through the respiratory chronic disease nursing clinic after being discharged from the hospital, including regular telephone or video consultations with specialist nurses. Patients could obtain personalized suggestions through remote communication and solve practical problems encountered in the rehabilitation process in a timely manner.

In addition, to reduce the potential influence of self-initiated rehabilitation behaviors, follow-up staff inquired during scheduled phone calls whether patients in the control group had initiated any structured exercise or pulmonary rehabilitation activities. No participants in the control group reported engaging in formal or systematic rehabilitation programs beyond the routine health education provided.

Through the above measures, it was ensured that patients in the control group could obtain sufficient educational support and standardized health management during routine care after being discharged from the hospital. This provided a stable control baseline for the study intervention group and helped analyze the actual effect of the home-based pulmonary rehabilitation program.

### 2.3 Observation indicators

#### 2.3.1 Primary observation indicators

(1) Quality of life: The Quality of Life in Bronchiectasis (QoL-B) questionnaire was adopted, which covered physiological function, role function, vitality, emotion, social function, treatment burden, sense of health and respiratory symptoms, etc. (2) Pulmonary function: FVC, FEV1, the ratio of FEV1/FVC and PEF were measured. It was ensured that the operation training before the tests was unified to make the data consistent.

#### 2.3.2 Secondary observation indicators

(1) Number of acute exacerbations: Record the frequency and nature (such as infection, dyspnea) of the need to seek medical treatment or be hospitalized due to aggravated symptoms. (2) Cough symptoms: Record the frequency and degree of cough.

### 2.4 Statistical methods

SPSS (Statistical Package for the Social Sciences) 26.0 software was used for statistical analysis to compare the differences between the two groups of patients in various observation indicators. For continuous variable data, a normality test was first carried out. If the preconditions such as normal distribution and homogeneity of variance were met, the two independent samples *t*-test was adopted. If the data did not conform to the normal distribution, the Mann–Whitney U test was used. For categorical variable data, the chi-square test was adopted. All statistical tests were two-sided, with the significance level set at *α* = 0.05. A *p* value less than 0.05 was considered to indicate a statistically significant difference. Statistical significance was determined based on a 95% confidence interval (CI).

## 3 Results

### 3.1 Baseline characteristics and intervention compliance

Among the 80 patients who completed the follow-up, there were 40 cases in the control group and 40 cases in the intervention group. Patients in the intervention group completed an average of 5.4 exercise sessions per week, with a median training duration of 45 min per session. The average Borg dyspnea score during training was 3.2. No safety-related adverse events were observed during the intervention period. There was no statistically significant difference in each clinical baseline data between the two groups of patients (*p* > 0.05). Details are shown in Table 1.

**Table 1:** Comparison of general information of the two groups of patients [(***x–*** ± s), number of cases (%)].

Project	Intervention group (*n* = 40)	Control group (*n* = 40)	Z/*t*/*χ^2^*	*P*
Gender			0.464^a^	0.496
Male	25 (62.50)	22 (55.00)		
Female	15 (37.50)	18 (45.00)		
Age (years)	57.48 ± 14.19	60.80 ± 13.06	1.089^b^	0.28
BMI (kg/m^2^)	20.96 ± 2.96	21.07 ± 3.15	0.161^b^	0.873
Educational attainment			0.207^c^	0.902
Junior high school or below	18 (45.00)	16 (40.00)		
Senior high school or technical secondary school	13 (32.50)	14 (35.00)		
College degree or above	9 (22.50)	10 (25.00)		
Medical insurance type			–	0.951
Urban medical insurance	36 (90.00)	35 (87.50)		
Rural cooperative medical insurance	1 (2.50)	2 (5.00)		
Public-funded medical insurance	1 (2.50)	1 (2.50)		
Self-paid	2 (5.00)	2 (5.00)		
Marital Status			–	0.644
Married	38 (95.00)	37 (92.50)		
Unmarried	2 (5.00)	3 (7.50)		
Self-care ability in daily life			–	0.396
Fully self-care	38 (95.00)	36 (90.00)		
Partially self-care with assistance	2 (5.00)	4 (10.00)		
Average monthly household income			1.755^c^	0.625
<3,000 yuan	7 (17.50)	12 (30.00)		
3,000 ~ 4,999 yuan	15 (37.50)	13 (32.50)		
5,000 ~ 9,999 yuan	14 (35.00)	12 (30.00)		
>10,000 yuan	4 (10.00)	3 (7.50)		
Exercise status			1.317^a^	0.251
Yes	22 (55.00)	27 (67.50)		
No	18 (45.00)	13 (32.50)		

Income brackets are presented in Chinese yuan (CNY) as reported by participants. To facilitate international interpretation, readers may refer to the World Bank’s global income classification based on gross national income (GNI) per capita for 2023: https://ourworldindata.org/grapher/world-bank-income-groups. Statistical tests: ^a^: Chi-square; ^b^: t-test; ^c^: Mann–Whitney.

### 3.2 Comparison of cough score (LCQ), number of acute exacerbations and pulmonary function indicators between the intervention group and the control group at different time points

There was no significant difference in the LCQ scores of the two groups of patients before the intervention and 1 month after the intervention (*p* > 0.05). Three months after the intervention, the LCQ score of the intervention group was 13.44 (11.75, 15.10), which was significantly higher than that of the control group [11.79 (8.67, 13.65)] (*p* = 0.005). Six months and twelve months after the intervention, the LCQ scores of the intervention group were 16.10 (15.58, 16.69) and 18.30 (17.81, 18.67), respectively, while those of the control group were 12.24 (11.33, 13.00) and 13.43 (13.00, 14.01), respectively, and the differences were all statistically significant (*p* < 0.001). There was no significant difference in the number of acute exacerbations before the intervention and 1 month after the intervention (*p* > 0.05). At 3 months, 6 months and 12 months after the intervention, the number of acute exacerbations in the intervention group was 1 (0, 1), 1 (0, 1) and 0.5 (0, 1), respectively, which were all significantly lower than that of the control group 2 (1, 2), 1 (1, 2) and 1 (0, 2), respectively, and the differences were statistically significant (*p* < 0.01). There was no significant difference in the pulmonary function indicators (FEV1, FVC, FEV1/FVC, PEF) between the two groups of patients before the intervention and one month after the intervention (*p* > 0.05). Three months after the intervention, the FEV1 of the intervention group was 2.25 ± 0.30 L, which was significantly higher than that of the control group (2.10 ± 0.32 L) (*p* = 0.034); the FVC was 2.92 ± 0.24 L, higher than that of the control group (2.84 ± 0.28 L) (*p* = 0.043); the FEV1/FVC was 76.01 ± 4.12%, higher than that of the control group (73.94 ± 4.07%) (*p* = 0.027); the PEF was 3.45 ± 0.43 L/s, higher than that of the control group (2.95 ± 0.46 L/s) (*p* < 0.001). Six months and twelve months after the intervention, the FEV1 of the intervention group was 2.38 ± 0.36 L and 2.56 ± 0.48 L respectively, which were significantly higher than that of the control group (2.14 ± 0.43 L and 2.20 ± 0.46 L respectively) (*p* < 0.01); the FVC was 3.10 ± 0.27 L and 3.16 ± 0.23 L, respectively, higher than that of the control group (2.88 ± 0.21 L and 2.93 ± 0.29 L respectively) (*p* < 0.001); the FEV1/FVC was 80.00 ± 4.17% and 81.01 ± 4.34% respectively, higher than that of the control group (74.31 ± 4.20% and 75.08 ± 4.19% respectively) (*p* < 0.001); the PEF was 4.09 ± 0.65 L/s and 4.68 ± 0.61 L/s respectively, higher than that of the control group (3.27 ± 0.59 L/s and 3.54 ± 0.53 L/s respectively) (*p* < 0.001) (Table 2).

**Table 2:** Comparison of cough conditions, pulmonary function indicators and the number of acute exacerbations of the two groups of patients at different time points [(***x–*** ± s), number of cases (%)].

		Intervention group (*n* = 40)	Control group (*n* = 40)	*t/Z*	*P*
Leicester Cough Questionnaire (LCQ) scores	Before intervention	8.91 (5.93, 12.10)	9.50 (6.60, 12.04)	−0.457	0.648
	One month after intervention	10.85 (9.12, 13.05)	10.19 (7.52, 13.12)	−0.803	0.422
	Three months after intervention	13.44 (11.75, 15.10)	11.79 (8.67, 13.65)	−2.834	0.005
	Six months after intervention	16.10 (15.58, 16.69)	12.24 (11.33, 13.00)	−7.38	<0.001
	Twelve months after intervention	18.30 (17.81, 18.67)	13.43 (13.00, 14.01)	−7.698	<0.001
The number of acute exacerbations	Before intervention	2 (1, 3)	2 (1, 2)	−0.333	0.739
	One month after intervention	2 (2, 3)	2 (1, 2)	−0.521	0.603
	Three months after intervention	1 (0, 1)	2 (1, 2)	−2.956	0.003
	Six months after intervention	1 (0, 1)	1 (1, 2)	−2.981	0.003
	Twelve months after intervention	0.5 (0, 1)	1 (0, 2)	−3.025	0.002
FEV1 (L)	Before intervention	2.01 ± 0.38	2.03 ± 0.39	0.232	0.817
	One month after intervention	2.04 ± 0.39	2.05 ± 0.41	0.112	0.911
	Three months after intervention	2.25 ± 0.30	2.10 ± 0.32	2.163	0.034
	Six months after intervention	2.38 ± 0.36	2.14 ± 0.43	2.707	0.008
	Twelve months after intervention	2.56 ± 0.48	2.20 ± 0.46	3.425	0.001
FVC (L)	Before intervention	2.78 ± 0.48	2.81 ± 0.43	0.294	0.769
	One month after intervention	2.81 ± 0.32	2.82 ± 0.35	0.133	0.894
	Three months after intervention	2.92 ± 0.24	2.84 ± 0.28	2.058	0.043
	Six months after intervention	3.10 ± 0.27	2.88 ± 0.21	4.363	<0.001
	Twelve months after intervention	3.16 ± 0.23	2.93 ± 0.29	3.93	<0.001
FEV1/FVC (%)	Before intervention	72.43 ± 7.30	72.35 ± 8.45	0.045	0.964
	One month after intervention	72.60 ± 5.48	72.70 ± 5.53	0.081	0.936
	Three months after intervention	76.01 ± 4.12	73.94 ± 4.07	2.261	0.027
	Six months after intervention	80.00 ± 4.17	74.31 ± 4.20	6.08	<0.001
	Twelve months after intervention	81.01 ± 4.34	75.08 ± 4.19	6.217	<0.001
PEF (L/s)	Before intervention	2.80 ± 0.49	2.78 ± 0.47	0.186	0.853
	One month after intervention	2.86 ± 0.52	2.83 ± 0.50	0.263	0.793
	Three months after intervention	3.45 ± 0.43	2.95 ± 0.46	5.022	<0.001
	Six months after intervention	4.09 ± 0.65	3.27 ± 0.59	5.908	<0.001
	Twelve months after intervention	4.68 ± 0.61	3.54 ± 0.53	8.922	<0.001

### 3.3 Comparison of the scores of various items regarding quality of life between the intervention group and the control group at different time points

The scores of each domain of QoL-B in both the intervention group and the control group basically conformed to the normal distribution, and the variances were homogeneous. The results of the independent sample *t*-test analysis showed that there was no statistically significant difference in the scores of each domain of QoL-B between the intervention group and the control group before the intervention and 1 month after the intervention (*p* > 0.05). Compared with the control group at 3, 6, and 12 months after the intervention, the scores of the intervention group in the domains of physical, emotional, role, and social functions, health perception, vitality, treatment impact, and respiratory symptoms of QoL-B were all higher, and the differences were all statistically significant (*p* < 0.05). The results of the repeated measures analysis of variance showed that in the domains of physical, emotional, and social functions of QoL-B, as well as the domains of health perception, vitality, treatment impact, and respiratory symptoms, the differences in time effect, between-group effect, and interaction effect were all statistically significant (*p* < 0.05). In the domain of role function, the differences in time effect and interaction effect were statistically significant (*p* < 0.05), while the difference in between-group effect was not statistically significant (*p* > 0.05). For details, please refer to Tables 3, 4.

**Table 3:** Comparison of various quality-of-life scores of the two groups of patients at different time points (***x–*** ± s).

Project	Time point	Intervention group (*n* = 40)	Control group (*n* = 40)	*t*	*P*
Physical function	Before intervention	65.25 ± 11.41	64.17 ± 11.87	0.415	0.679
	One month after intervention	70.59 ± 9.46	67.36 ± 10.42	1.452	0.151
	Three months after intervention	79.48 ± 6.38	71.11 ± 7.93	5.201	<0.001
	Six months after intervention	85.30 ± 4.30	76.32 ± 5.32	8.303	<0.001
	Twelve months after intervention	89.35 ± 3.10	80.46 ± 6.31	7.997	<0.001
Health perception	Before intervention	54.33 ± 14.12	53.15 ± 14.87	0.364	0.717
	One month after intervention	58.65 ± 12.36	56.27 ± 12.46	0.858	0.394
	Three months after intervention	67.68 ± 10.21	60.78 ± 10.89	2.923	0.005
	Six months after intervention	75.52 ± 7.13	65.45 ± 10.37	5.061	<0.001
	Twelve months after intervention	88.22 ± 3.92	78.58 ± 5.68	8.834	<0.001
Vitality	Before intervention	55.25 ± 14.33	56.13 ± 14.30	0.275	0.784
	One month after intervention	59.85 ± 9.87	58.42 ± 9.15	0.672	0.504
	Three months after intervention	68.37 ± 8.91	61.29 ± 8.78	3.58	0.001
	Six months after intervention	76.58 ± 6.84	66.37 ± 8.16	6.065	<0.001
	Twelve months after intervention	85.31 ± 4.45	74.57 ± 5.28	9.837	<0.001
Emotional function	Before intervention	60.42 ± 10.45	59.76 ± 12.53	0.256	0.799
	One month after intervention	62.05 ± 8.29	61.07 ± 9.31	0.497	0.62
	Three months after intervention	71.53 ± 6.49	64.94 ± 8.61	3.866	<0.001
	Six months after intervention	79.09 ± 6.33	71.13 ± 6.35	5.615	<0.001
	Twelve months after intervention	89.35 ± 3.34	78.76 ± 5.13	10.941	<0.001
Treatment impact	Before intervention	53.25 ± 15.30	52.13 ± 15.32	0.327	0.744
	One month after intervention	55.85 ± 13.76	55.42 ± 13.19	0.143	0.887
	Three months after intervention	67.37 ± 10.72	59.29 ± 10.75	3.366	0.001
	Six months after intervention	75.58 ± 7.68	64.37 ± 9.37	5.852	<0.001
	Twelve months after intervention	82.28 ± 5.28	73.59 ± 6.35	6.655	<0.001
Role function	Before intervention	59.64 ± 12.23	58.47 ± 13.32	0.409	0.684
	One month after intervention	62.03 ± 9.52	60.28 ± 9.39	0.828	0.41
	Three months after intervention	68.85 ± 7.26	64.62 ± 8.16	2.449	0.017
	Six months after intervention	79.48 ± 5.54	73.60 ± 6.13	4.501	<0.001
	Twelve months after intervention	87.26 ± 4.23	79.55 ± 5.16	7.308	<0.001
Social function	Before intervention	60.27 ± 13.18	61.22 ± 13.12	0.323	0.748
	One month after intervention	63.24 ± 10.15	62.21 ± 11.13	0.432	0.667
	Three months after intervention	69.22 ± 8.12	64.24 ± 9.10	2.582	0.012
	Six months after intervention	79.23 ± 6.11	73.25 ± 7.15	4.021	<0.001
	Twelve months after intervention	89.65 ± 3.91	80.38 ± 5.79	8.392	<0.001
Respiratory symptoms	Before intervention	55.73 ± 15.40	56.23 ± 15.20	0.146	0.884
	One month after intervention	59.92 ± 13.78	58.07 ± 13.18	0.614	0.541
	Three months after intervention	69.35 ± 8.29	61.13 ± 9.05	4.236	<0.001
	Six months after intervention	78.26 ± 7.74	68.10 ± 8.21	5.695	<0.001
	Twelve months after intervention	89.81 ± 3.60	75.76 ± 5.53	13.467	<0.001

**Table 4:** Repeated-measures analysis of variance of quality-of-life scores of two groups of patients at different time points.

QoL-B domain	Between-group effect		Time effect		Interaction effect	
	*F*	*P*	*F*	*P*	*F*	*P*
Physical function	4.086	0.003	81.950	<0.001	55.810	<0.001
Health perception	2.893	0.022	94.900	<0.001	31.460	<0.001
Vitality	6.008	<0.001	83.400	<0.001	35.960	<0.001
Emotional function	5.864	<0.001	120.800	<0.001	43.750	<0.001
Treatment impact	3.659	0.006	67.030	<0.001	27.300	<0.001
Role function	2.054	0.086	116.300	<0.001	23.320	<0.001
Social function	3.840	0.005	96.240	<0.001	19.210	<0.001
Respiratory symptoms	6.228	<0.001	82.490	<0.001	39.550	<0.001

## 4 Discussion

This study explored the application effect of the evidence-based home-based pulmonary rehabilitation program (HBPR) among discharged patients with bronchiectasis. The results demonstrated that, compared with routine care, HBPR significantly improved patients’ quality of life, pulmonary function indicators, and exercise endurance, and also reduced the number of acute exacerbations. These findings provided important evidence for the long-term management of bronchiectasis and laid a foundation for promoting the application of home-based rehabilitation in chronic respiratory diseases.

### 4.1 Multidimensional improvement of home-based pulmonary rehabilitation on quality of life

The exploratory analysis of this study revealed that at 3, 6, and 12 months after the intervention, patients in the intervention group scored significantly higher than those in the control group across all domains of the QoL-B questionnaire, including physical functioning, emotional functioning, role functioning, social functioning, health perceptions, vitality, treatment burden, and respiratory symptoms (*p* < 0.05). Repeated measures analysis of Variance (ANOVA)further confirmed that significant time effects, group effects, and interaction effects were observed in the domains of physical, emotional, social functioning, health perception, vitality, treatment burden, and respiratory symptoms (*p* < 0.05). For the role functioning domain, significant time and interaction effects were also identified (*p* < 0.05). These findings are consistent with the randomized controlled trial conducted by Araújo et al. on COPD patients, which demonstrated that pulmonary rehabilitation can significantly improve quality of life by enhancing exercise capacity and alleviating symptom burden (7).

From the perspective of intervention mechanisms, the observed improvements likely resulted from the synergistic effects of multiple components. First, exercise training enhanced skeletal muscle oxidative capacity and muscular endurance, playing a central role in improving physical function and promoting independence in daily activities (8). Second, airway clearance techniques, such as postural drainage and active breathing techniques, helped reduce mucus retention, lower the risk of recurrent infections, and improve airway patency, thereby alleviating dyspnea and fatigue (9). On the psychological and social levels, health education and remote follow-up feedback systems strengthened patients’ sense of disease control, increased adherence, and fostered psychological resilience (10).

It is noteworthy that the HBPR program implemented in this study incorporated a phased training design, personalized intensity adjustment, and multimodal supervision-feedback mechanisms, forming a closed-loop intervention model. These features helped overcome the limitations of traditional passive educational interventions. Although the control group also received standard respiratory care—including educational brochures, online video resources, and follow-up consultations—such interventions were largely passive and lacked individualized supervision or progressive programming. While traditional approaches may improve basic disease awareness and promote limited behavioral changes, they remain inadequate in maintaining long-term engagement and delivering measurable improvements in pulmonary outcomes. Internationally, Benzo et al. (11) found that COPD patients who underwent 12 weeks of remote monitoring and health coaching showed significant improvements in health-related quality of life (measured by Chronic Respiratory Questionnaire (CRQ)), including dyspnea, fatigue, emotional function, exercise capacity, and daily activities, with sustained effects at 24 weeks. Chapman et al. (12) also demonstrated that structured and systematic rehabilitation interventions can effectively improve psychological well-being in patients with bronchiectasis. These studies further support the rationale and implementation of the HBPR model in our study. Compared with the traditional hospital rehabilitation model, home-based pulmonary rehabilitation offers greater flexibility in time and resources, providing a sustainable long-term intervention approach for patients with chronic diseases.

It is worth emphasizing that although patients in the control group received routine respiratory care, including educational pamphlets, online video resources, and follow-up consultations, these measures were largely passive and lacked personalized supervision or structured progression. While such conventional approaches may enhance basic disease awareness and encourage limited behavioral adjustments, their effectiveness in maintaining long-term engagement and achieving measurable improvements in lung health remains limited. In contrast, the HBPR program in our study offered a multidimensional, actively monitored intervention that incorporated physical training, breathing techniques, nutritional support, and real-time feedback, contributing to significantly superior outcomes. This highlights the limitations of standard care and supports the need for structured and individualized rehabilitation models in the management of bronchiectasis.

### 4.2 Analysis of the improvement and mechanism of pulmonary function indicators

The pulmonary function indicators of the patients in the intervention group, including FEV₁ (Forced Expiratory Volume in the First Second), FVC (Forced Vital Capacity), PEF (Peak Expiratory Flow), etc., all showed significant improvement during the follow-up period, especially after 6 months and 12 months. For example, the FEV₁ in the intervention group significantly increased from the baseline of 1.12 ± 0.15 L to 1.35 ± 0.18 L at 6 months (*p* < 0.001), and further increased to 1.40 ± 0.17 L at 12 months (*p* < 0.001). This result is consistent with the research on patients with chronic obstructive pulmonary disease (COPD) conducted by McCarthy et al. They pointed out that long-term pulmonary rehabilitation interventions can effectively improve pulmonary function and slow down the rate of decline in pulmonary function (13). The mechanism may be related to the combined effect of inspiratory muscle training and aerobic exercise. Inspiratory muscle training can improve the efficiency of alveolar ventilation by strengthening the strength and endurance of the respiratory muscles. Aerobic exercise reduces the peripheral airway resistance and improves dynamic pulmonary hyperinflation (14). Secondly, airway clearance techniques can further reduce the burden of airway inflammation and improve pulmonary function by reducing the accumulation of airway secretions (15). Furthermore, nutrition management ensures that patients receive adequate nutritional support. Health education promotes the formation of healthy behaviors among patients. Quality management, through remote technology monitoring and guidance, ensures the safety and effectiveness of the training. These measures work together and contribute to the improvement of patients’ pulmonary function. The results of this study further verify the effectiveness of HBPR in patients with bronchiectasis, indicating that this program is not only effective for symptom management but also can fundamentally improve the state of pulmonary function.

### 4.3 The impact of home-based pulmonary rehabilitation on cough symptoms

Relevant investigations have shown (16) that among patients with bronchiectasis, those with chronic cough symptoms account for 82–98%, and chronic cough is significantly correlated with an increased symptom burden of bronchiectasis, an increased disease severity, and an increased frequency of exacerbations. The results of this study demonstrated that compared with the control group at 3, 6, and 12 months after the intervention, the scores of the Leicester Cough Questionnaire (LCQ) of the patients in the intervention group were all higher, and the differences were all statistically significant (*p* < 0.05). It indicates that the HBPR program constructed in this study has a long-term and positive impact on improving the chronic cough symptoms of patients with bronchiectasis. The possible reason is that the exercise training in the HBPR program can enhance cardiopulmonary function and reduce dyspnea, thereby preventing the occurrence of cough and expectoration. Airway clearance therapy helps increase the airway flow velocity and improve the fluidity of sputum through physical therapies such as promoting effective coughing, huffing, vibration, and chest percussion, as well as guiding patients to perform specific breathing techniques, such as pursed-lip breathing and abdominal breathing. In this way, it can help patients loosen the viscous secretions from the airway walls and expel them out of the body, so as to clear the sputum that may lead to bacterial reproduction and inflammatory reactions, reduce the risks of airway obstruction and bacterial infection, and relieve the symptoms of cough and expectoration caused by airway irritation (17). Nutrition management improves the overall nutritional status of patients, enhances their immunity, and reduces the probability of infection. Health education improves patients’ understanding of the disease and their ability to cope with it, which is conducive to symptom control and thus continuously improves the symptoms of cough and expectoration (18).

### 4.4 Reduction in the number of acute exacerbations and its clinical significance

The study found that the number of acute exacerbations in the intervention group was significantly reduced, indicating that HBPR plays an important role in reducing the risk of disease deterioration. The application of airway clearance techniques may be one of the key factors. It significantly reduces infection-related acute exacerbations by clearing sputum and reducing the risk of airway bacterial colonization (19). In addition, health education has improved patients’ ability to identify acute exacerbations early and respond in a timely manner, thereby reducing the incidence of severe acute exacerbations (20). This finding is consistent with the research on patients with bronchiectasis conducted by Lee et al. They emphasized the remarkable effect of multidimensional rehabilitation interventions in reducing disease deterioration (21). For patients with chronic diseases, reducing acute exacerbations can not only improve patients’ quality of life but also significantly reduce the use of medical resources, demonstrating the economic value of this intervention program.

Meanwhile, the results of this study show that HBPR, as a low-cost and high-compliance intervention model, has significant advantages in improving the quality of life of patients with bronchiectasis and reducing the disease burden. Its personalized and convenient features make it particularly suitable for regions with limited medical resources. Compared with the traditional inpatient rehabilitation model, HBPR can significantly improve patients’ intervention compliance through remote guidance and family support, while reducing patients’ dependence on medical institutions and having high sustainability (22). In addition, through the application of the e-health platform, this program can also monitor patients’ rehabilitation progress in real time and further optimize the intervention strategies. These features make HBPR an innovative intervention method that meets the needs of modern medicine.

### 4.5 Research deficiencies and future prospects

Although this study provides preliminary evidence supporting the effectiveness of HBPR in patients with stable bronchiectasis, several limitations should be noted. First, the relatively small sample size may limit the generalizability of the findings, and larger multicenter trials are needed for further validation. Second, the 12-month follow-up period may not fully capture the long-term sustainability or prognostic impact of the intervention. Third, although baseline characteristics such as age and lung function were balanced between groups, this study did not stratify patients based on standardized severity scores such as FACED (FEV₁, Age, Chronic colonization, Extension, and Dyspnea) or the Bronchiectasis Severity Index (BSI), nor did it systematically record disease etiology, both of which may influence treatment response and prognosis. Finally, while the study focused primarily on physiological outcomes such as pulmonary function and quality of life, future studies should incorporate broader dimensions such as mental health, self-efficacy, and social adaptability to better reflect patient-centered outcomes.

## 5 Conclusion

This study demonstrates that the evidence-based HBPR is an effective intervention measure that can significantly improve the quality of life, pulmonary function, and clinical outcomes of patients with bronchiectasis and reduce the number of acute exacerbations. Its low cost, high compliance, and convenience provide important implications for the management of chronic respiratory diseases. Specifically, HBPR can be promoted in environments with limited medical resources as a complementary or alternative strategy to inpatient rehabilitation. Meanwhile, future studies should further optimize the intervention content, such as personalized exercise prescriptions and remote monitoring technologies, to enhance the implementation effect and expand its scope of application. In addition, exploring longer-term follow-up data will help to evaluate the broad applicability of HBPR among different populations and provide a scientific basis for the standardized management of chronic respiratory diseases.

## Data Availability

The raw data supporting the conclusions of this article will be made available by the authors, without undue reservation.
